# Coexistence of CN1A autoantibodies in GAD65 encephalitis exacerbates neurodegeneration: Novel autoantibodies in GAD65 encephalitis

**DOI:** 10.1186/s12974-025-03521-4

**Published:** 2025-07-26

**Authors:** Annika Breuer, Delara Kamalizade, Tobias Baumgartner, Juliane L. Berns, Thoralf Opitz, Franziska S. Thaler, Susanne Schoch, Lars Komorowski, Christoph Helmstaedter, Rainer Surges, Albert J. Becker, Julika Pitsch

**Affiliations:** 1 Department of Epileptology, Medical Faculty, University of Bonn, Venusberg-Campus 1, D-53127 Bonn, Germany; 2 Inst. of Experimental Epileptology and Cognition Research, University Hospital Bonn, Bonn, Germany; 3 Inst. of Clinical Neuroimmunology, LMU University Hospital, LMU Munich, Munich, Germany; 4 Biomedical Center (BMC), Faculty of Medicine, LMU Munich, Munich, Germany; 5 Institute of Cellular Neurosciences II, University Hospital Bonn, Bonn, Germany; 6 Institute of Experimental Immunology, EUROIMMUN AG, Lübeck, Germany

**Keywords:** Glutamate decarboxylase 65 (GAD65), Cytosolic 5'-nucleotidase 1A (CN1A), Temporal lobe epilepsy, Limbic encephalitis, Autoimmune encephalitis

## Abstract

**Background:**

Autoantibodies targeting the intracellular 65-kDa isoform of glutamic acid decarboxylase (anti-GAD65) have been associated with a variety of autoimmune-related syndromes involving a spectrum of difficult-to-treat neurological disorders. However, the pathophysiological role of anti-GAD65 in neuroinflammation remains vague. Its understanding may be complicated by the possible pathogenic interaction between anti-GAD65 and potentially coexisting autoantibodies.

**Methods:**

We combined a broad spectrum of approaches ranging from antibody-antigen identification, immunoblotting, immunoprecipitation, mass-spectrometry, cell-based assays, subcellular binding pattern analysis in primary neuronal cultures, and immunohistochemistry to in vitro assays of neuronal uptake, viability, and multi-electrode arrays.

**Results:**

In anti-GAD65-positive neurological patients, mass-spectrometric analysis revealed cytosolic 5’-nucleotidase 1 A (CN1A syn. NT5C1A) as the most abundant antigen. Subsequent screening of 118 anti-GAD65-positive patients revealed that 32 of them had additional autoantibodies targeting CN1A, which were also present in all available corresponding CSF samples. Limbic encephalitis was more often diagnosed in anti-CN1A/anti-GAD65-positive compared to the anti-GAD65-positive patients. Functionally, incubation of primary hippocampal neurons with anti-GAD65, but not with anti-CN1A, resulted in uptake into GABAergic neurons, neuronal cell death, and increased neuronal network activity. Moreover, simultaneous incubation with both antibodies (anti-CN1A/anti-GAD65) resulted in concomitant intraneuronal uptake in a concentration-dependent manner, which correlated with enhanced autophagy followed by massive neuronal death.

**Conclusion:**

GAD65 antibodies directly affect neuronal viability and network activity. Co-existing autoantibodies against CN1A, present in anti-GAD65-positive patients, enhance autophagy and subsequent neuronal death in vitro. Clinically, anti-GAD65-positive patients should be screened for anti-CN1A-associated diseases, and evaluation of anti-CN1A in anti-GAD65-related autoimmune conditions may clarify links between systemic autoimmunity and epilepsy.

**Supplementary Information:**

The online version contains supplementary material available at 10.1186/s12974-025-03521-4.

## Significance

**What is already known on this topic**– Even though autoantibodies against GAD65 protein have been identified in several autoimmune disorders their direct pathogenic effects are highly controversial and patients are often difficult to treat.

**What this study adds**– Anti-GAD65-positive patients often have coexisting CN1A autoantibodies, leading to higher incidence of limbic encephalitis, increased neuronal activity, with combined autoantibody uptake enhancing neuronal death rate correlated with enhanced autophagy.

**How this study might affect research**,** practice or policy**– Patients positive for anti-GAD65 should be screened for anti-CN1A-associated diseases, and broader evaluation of anti-CN1A in anti-GAD65-related autoimmune conditions (e.g., type 1 diabetes, vitiligo, Hashimoto’s thyroiditis) may clarify links between systemic autoimmunity and epilepsy.

**Supplementary Information:**

The online version contains supplementary material available at 10.1186/s12974-025-03521-4.

## Bullet points

Anti-GAD65-positive neurological patients show a high prevalence of coexisting autoantibodies against CN1A.

CN1A/GAD65 autoantibody-positive patients are more often diagnosed with limbic encephalitis.

Patient-derived GAD65 autoantibodies increase network activity in cultured neurons.

Interneuronal dendritic uptake of anti-CN1A combined with anti-GAD65 leads to aggravated neurodegeneration associated with enhanced autophagy.

**Supplementary Information:**

The online version contains supplementary material available at 10.1186/s12974-025-03521-4.

## Introduction

The 65-kDa isoform of glutamic acid decarboxylase (GAD65) is critical for the rapid synthesis and conversion of glutamate to gammaaminobutyric acid (GABA), the major inhibitory neurotransmitter of the CNS and thus required for synaptic transmission. To date, autoantibodies targeting GAD65 have been associated with neurological autoimmune-related syndromes in adults, including cerebellar ataxia, stiff-person syndrome, and drug-resistant TLE [1, 2]. The latter may emerge as a chronic stage of GAD65 autoantibody-associated limbic encephalitis (LE) [3]. Pathogenic cerebral T-cell infiltration [4] and a regulatory effect of CD4^+^ T-cells [5, 6] are observed resulting in progressive focal or generalised brain atrophy leading to pharmacoresistant seizures and prolonged memory impairment with advancing disease [7]. Nevertheless, limited response to immunotherapy already at early disease stages is detected in anti-GAD65 encephalitis. Interestingly, autoantibodies targeting GAD65 are also valuable biomarkers for the diagnosis of type 1 diabetes mellitus (T1DM), as they are present in 80% of these patients, but generally at much lower levels [8]. Although the clinical range of “GAD antibody spectrum disorders” has greatly expanded and has shown increasing heterogeneity [9], the reasons for this diversity remain unclear. Potential explanations include the differential susceptibility of GABAergic neurons to anti-GAD65, differences in epitope specificity or other unidentified coexisting autoantibodies [10]. The coexistence of GAD65 autoantibodies with neuronal surface autoantibodies and autoantibodies targeting intracellular antigens has been reported but is rare [11]. Importantly, the clinical relevance of low-titre GAD65 antibodies is considerably lower compared to high-titre antibodies, particularly in patients presenting with neurological symptoms. The definition of ‘low’ versus ‘high’ titre is method-dependent, as different assays (e.g., RIA, ELISA, CBA, line blot) have varying sensitivity and thresholds for detection [12, 13]. High-titre GAD65 positivity, especially when coupled with CSF positivity, is more strongly associated with neurological disease, whereas low-titre positivity is often seen in patients with T1DM or even healthy controls [14]. Therefore, the clinical significance of GAD65 autoantibodies should be taking into account both the detection method and the presence of neurological symptoms.

Immediate pathogenic effects of autoantibodies against GAD65 protein are highly controversial, partially because of the intracellular localisation of the target antigen [4]. While some data suggests that GAD65 autoantibodies may be a non-pathogenic epiphenomenon of autoimmune diseases [13, 15], they are nonetheless valuable as biomarkers for inflammatory processes [4]. Conversely, other studies indicate that anti-GAD65 antibodies may directly impair GABAergic synaptic transmission [16] and inhibit GAD65 enzymatic activity, thus blocking the formation and function of GABA [17].

In the present study, we investigate the potential pathogenic role of patient-derived GAD65 autoantibodies associated with neurological diseases. We also analyse in detail the presence of coexisting autoantibodies and their possible functional interactions with anti-GAD65 to understand, whether such coexistence modifies pathogenicity in this clinically heterogeneous patient group.

## Patients, materials and methods

### Standard protocol approvals, registrations, and patient consents

All procedures were conducted in accordance with the principles of Declaration of Helsinki. Informed written consent was obtained from every patient according to the approvals of the local ethics committee of the University Hospital Bonn, Bonn, Germany (Nr. 222/16, 229/18, 371/20, 504/20). All animal procedures were planned and performed to minimize pain and suffering, and to reduce the number of animals used in accordance with European, national, and institutional guidelines (European Directive 2010/63/EU; ARRIVE). The study protocol was approved by the Landesamt für Natur, Umwelt und Verbraucherschutz (LANUV) of the state of North Rhine Westphalia, Germany.

We confirm that we have read the Journal’s position on issues involved in ethical publication and affirm that this report is consistent with those guidelines.

### Autoantibody diagnostics

All analysed samples were screened for neurological autoantibodies previously associated with autoimmune encephalitis (AE) and other neuroimmunological syndromes, using commercial kits for diagnostic procedures (see supplement).

### Human samples

Neurological patients who tested positive for anti-GAD65 (n = 118) were analysed for the presence of coexisting autoantibodies (University Hospital Bonn: n = 98); Institute of Clinical Neuroimmunology Munich: n = 20). To test a broad spectrum of GAD65-positive patients, neurological patients with positively tested CSF and/or serum in either semi-quantitative immunoblots or cell-based assays were included. These results correspond to GAD65 antibody levels ≥ 1,400 U/ml or 1,800 U/ml, thresholds that by definition classify affected patients as ‘high titre’ anti-GAD65 seropositive [12, 13]. Additionally, sera of 32 neurological patients with specific onconeuronal autoantibodies, 97 age-/sex-matched seronegative neurological, and 60 healthy were analysed (supplement).

### Collection of clinical data

Relevant clinical information such as medication, age at disease onset, and comorbidities, including autoimmune disorders and malignancies was extracted retrospectively from detailed medical reports. Patients were classified based on their clinical presentation into LE, chronic temporal lobe epilepsy (TLE), Stiff-person syndrome, and cerebellar ataxia [1, 14]. Patients with seizures were retrospectively classified by two expert neurologists into chronic TLE or LE [18]. Accordingly, patients with (sub-)acute onset and significant cognitive deficits were classified as having LE [18], and patients with a more chronic onset were classified as having chronic TLE. Neuropsychological assessment included the domains of IQ, executive function, verbal and visual memory, and mood. The standardized neuropsychological tests have been described in detail elsewhere [19] and were conducted by neuropsychologists from the Department of Epileptology in Bonn.

### Screening for additional autoantibodies

For immunoblot screening, protein lysates of rat and mouse brains, and of murine crude synaptosomes were prepared as previously described [20]. For immunofluorescence screenings, we used formalin-fixed human hippocampal, cerebellar, and rat and mouse brain sections.

### Immunoprecipitation and mass spectrometry

Immunoprecipitation (IP) and protein identification mass spectrometry were performed as previously described [20, 21] with minor modifications (supplement).

### Validation of CN1A autoantibody binding

To develop screening assays for autoantibodies against cytosolic 5’-nucleotidase 1 A (CN1A), we performed immunoblotting with purified recombinant human CN1A protein. Additionally, a cell-based assay was established to validate the screening, as described in the supplementary material.

### Primary hippocampal neurons

Dissociated primary hippocampal neurons (PHN) of E15-19 murine hippocampi (C57Bl6/N) were prepared as described previously [21]. For the generation of one biological replicate, one litter of embryos was used. In line with 3R, generated primary neurons were used for several other approaches.

### Immunocytochemistry

PHN were transduced at day in vitro (DIV) 4–6 with rAAVs expressing fluorescent proteins in either excitatory or inhibitory neurons, fixed and further processed at DIV14 as described in the Supplement. Experiments were performed in four biological replicates consisting of four technical replicates each.

### Antibody uptake and neuronal viability assays

PHN were incubated with primary antibodies (rabbit anti-CN1A, PA5-101545, ThermoFisher; mouse anti-GAD65, ab26113, abcam) or control IgG in different combinations and concentrations (20 µg/ml or 10 µg/ml) for 4–24 h at 37 °C. The PHN were washed with PBS, fixed (4% PFA) for 10 min, and stained with the respective secondary antibodies and mounted as described above. Neuronal quantification was performed in four biological replicates consisting of four technical replicates each.

### Immunohistochemistry

Immunohistochemistry was performed on 4 μm thick paraffin sections of mouse brains using standard protocols [21], described in supplement.

### Measurement of network activity in hippocampal neurons

With multi-electrode arrays (MEA), network activity was recorded simultaneously (Maestro Edge, Axion Biosystems, Atlanta) as described previously comparing anti-CN1A/anti-GAD65, anti-CN1A/onconeuronal antibody (CV2, MA2, AMPH), anti-CN1A, or anti-GAD65-positive patients (pooled samples of *n* = 4). Data was recorded with AxIS software (1.5, Axion Biosystems, Atlanta).

### Autophagy approach

PHN were incubated on DIV13 with antibodies (rabbit anti-CN1A, PA5-101545, ThermoFisher; mouse anti-GAD65, ab26113, abcam) or control IgG (further processing see supplement). Autophagy was determined by measuring the average lysosomal size (area) in three biological replicates consisting of four technical replicates.

### Images

Images were taken using a laser-scanning Nikon A1/Ti confocal microscope with a PlanAPOIR60×WI objective (NA 1.27). Data were processed using Nikon NIS-Elements 4.0 acquisition software.

### Statistical analysis

Statistical comparisons of the clinical characteristics between CN1A/GAD65 and GAD65 antibody-positive groups were performed using Chi-square test including Yate’s correction, or Fisher’s exact test for comparing the two categorical variables. For multiple comparisons, p-values were adjusted by Bonferroni correction. Mann-Whitney U-test and Kruskal-Wallis with Dunn’s multiple comparisons test was used for nonparametric statistics. Two-way ANOVA with Tukey’s multiple comparisons test was used for parametric statistics. Neuropsychological data were compared between groups using ANOVA. Correlations were calculated using Pearson´s correlations. Values were considered significant at *p* < 0.05. All calculations were performed using GraphPadPRISM.

## Results

### Identification of a coexisting autoantibody in anti-GAD65-positive neurological patients

**Fig. 1:**
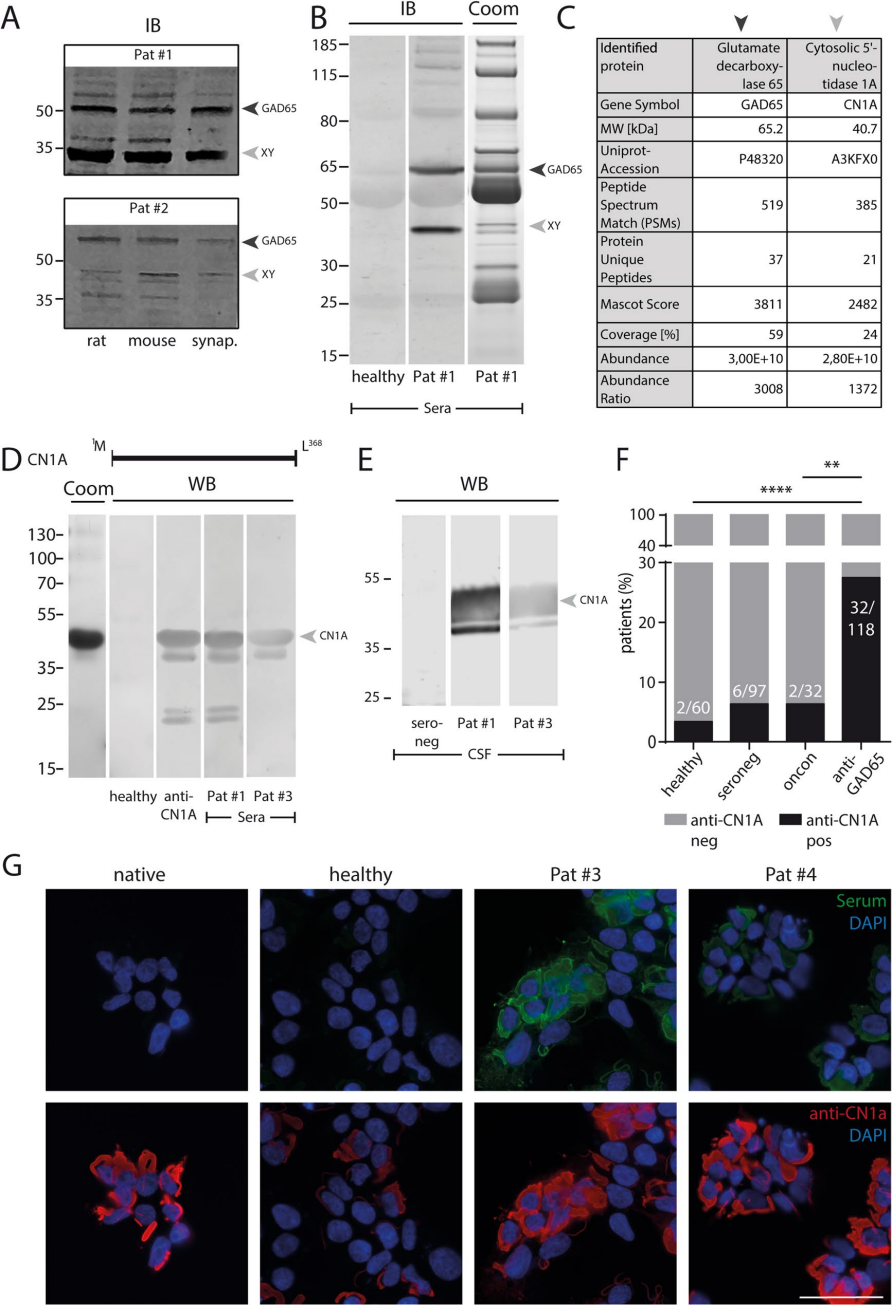
Identification of a coexisting autoantibody in GAD65 autoantibody positive neurological patients. (**A**) Immunoblot of rat, mouse and synaptosomal brain homogenates incubated with serum from two representative anti-GAD65-positive patients (Pat #1 and 2) shows strong bands at around 65 kDa (black arrow), indicating GAD65 antigen, and at a 40 kDa protein (“xy”, grey arrow), resembling an unknown antigen. (**B**) An immunoblot of an immunoprecipitation using the Pat #1 serum and whole brain lysate validates bands at the same size. Coomassie stained SDS-PAGE of whole brain lysate immunoprecipitated with patient and control sera from healthy individuals were excised at approximately 65 and 40 kDa and processed by mass spectrometry. (**C**) Mass spectrometry data analysis confirmed the presence of the target antigen GAD65 (black arrow) and identified the novel autoantibody target CN1A (grey arrow). (**D**) Scheme of the full-length CN1A protein (CN1A). Screening of anti-GAD65-positive sera for the coexistence of CN1A autoantibodies revealed reactivity against purified full-length CN1A protein in immunoblots of sera from two representative GAD65-positive patients (Pat #1 and 2). Coomassie Brilliant Blue staining shows the amount of purified protein loaded for immunoblotting. (**E**) Representative immunoblots of the two positive patients. (**F**) Distribution of CN1A autoantibody positivity in different patient cohorts (2/60 healthy control, 6/97 seronegative patients, 2/32 onconeuronal (oncon) autoantibody positive sera (including MA2: 1/14, CV2 1/11, AMPH: 0/7), 32/118 GAD65 autoantibody positive sera; Fisher’s exact test with Bonferroni correction: healthy vs. seronegative: *p* = 0.711; healthy vs oncon *p* = 0.619; healthy vs. GAD65: **** *p* < 0.0001; oncon vs. GAD65: **0.0091). (**G**) Representative images of cell-based assay of a native culture, a negative serum sample of a healthy individual, and two positive patients. Co-staining with commercial anti-CN1a Abs indicates specific binding (scale bar: 50 μm). xy = unknown protein

To determine the potential prevalence of coexisting autoantibodies in anti-GAD65-positive neurological patients, samples from 118 patients were retrospectively analysed. Common neuronal and AE-associated onconeuronal autoantibodies were sporadically detected in this cohort (Table 1) using commercially available diagnostic kits. Of the anti-GAD65-positive sera tested in the unspecific screening for brain protein-reactivity (*n* = 98), 38% had either a neuropil (10%) or neuronal (28%) binding pattern on rat or mouse brain sections. Serum samples from 23% of GAD65-positive patients (23/98) showed additional bands of different sizes on immunoblots after incubation with rat and mouse brain homogenates or synaptosome fractions, additionally to the 65-kDa band, indicating additional antigen targets. In some patients, a strong band at ~ 40-kDa was detected (Fig. 1A; representative blots), that was absent in controls. IP was performed on serum samples from these patients incubated with brain lysate (Fig. 1B, representative blot patient #1). Subsequent mass spectrometric analysis revealed enriched protein concentrations of CN1A with a high abundance ratio and a significant Mascot score (molecular weight 40-kDa, Fig. 1C) in two anti-GAD65-positive index patients verified in two independent experiments, which were not present in any of the controls (*n* = 3 control patients with focal epilepsy without any signs of autoimmune encephalitis, *n* = 3 healthy controls).

**Table 1:** Clinical characteristics of neurological GAD65-positive patients

	CN1A/GAD65-positive (*n* = 32)	GAD65-positive (*n* = 86)	*p* value*
Age at onset (y), median (range)	43 (8–71)	44 (2–79)	0.2546
Female, n (%)	21 (65.6)	56 (65.1)	0.5697
Dominant neurological syndromes/symptom			
Limbic encephalitis (LE), n (%)	8 (25.0)	8 (9.3)	0.0320
Focal epilepsy, n (%)	17 (46.9)	51 (59.3)	0.1586
Cerebellar ataxia (CA), n (%)	1 (3.1)	9 (10.5)	0.1838
Stiff-person syndrome (SPS), n (%)	3 (9.4)	2 (2.3)	0.1198
Diverse/ unspecific syndromes, n (%)^a^	5 (15.6)	16 (18.6)	0.4580
Two overlapping syndromes, n (%)^b^	3 (9.4)	8 (9.3)	0.4952
Autoimmune comorbidity, n (%)	13 (40.6)	33 (38.4)	0.4928
Diabetes mellitus type 1, n (%)	8 (25.0)	12 (14.0)	0.1271
Autoimmune thyroiditis, n (%)	7 (21.9)	19 (22.1)	0.5966
Other, n (%)^c^	2 (6.3)	8 (9.3)	0.4574
Other autoantibodies (AB)			0.5829
Neuronal cell-surface AB, n (%)^d^	0 (0)	3 (3.5)	
Onconeuronal AB, n (%)^e^	3 (9.4)	6 (7.0)	
Immunotherapy (IT), n (%)	19 (61.3)	44 (52.4)	0.1542
Number of different ITs, median (range)^f^	1 (1–4)	2 (1–7)	0.1170
Symptom free with IT, n (%)	3 (21.4)	4 (10.5)	0.2754
Not symptom free with IT, n (%)	11 (78.6)	34 (89.5)	
lost in follow-up, n	6	6	
Seizures, n (%)^j^	27 (84.4)	63 (73.3)	0.1539
epilepsy, n (%)	25 (92.6)	58 (92.1)	0.6498
Pharmacoresistant epilepsy, n (%)^g^	14 (66.7)	39 (72.2)	0.4175
Seizure free with ASM, n (%)^h^	7 (33.3)	15 (27.8)	
lost in follow-up, n	4	4	
Tumors			
Non-CNS tumors, n (%)^i^	1 (3.1)	6 (7.0)	0.3873

* Statistical comparisons between CN1A/GAD65 and GAD65 antibody positive groups were performed using Chi-square test including Yates’s correction, and Fisher’s exact test for comparing the two categorical variables. Mann-Whitney U-test was used for nonparametric statistics

^a^ including cognitive impairments, polyneuropathy, single epileptic seizure, movement disorders, depression, undefined delir, Neuritis nervi optici, steroid-responsive encephalopathy associated with autoimmune thyroiditis, developmental disorder, affective disorder, idiopathic generalised epilepsy, epilepsy of unknown type

^b^ CN1A positive: LE + SPS (1), SPS + epilepsy (2); GAD65 positive: LE + depression (2), epilepsy + CA (1), epilepsy + SPS (1), epilepsy + anxiety/cognitive impairment (2), CA + cognitive impairment (1), SPS + affective disorder (1)

^c^ Multiple sclerosis, Crohn´s disease, vitiligo, Morbus Bechterew, Morbus Basedow, APECED syndrome, Asthma bronchiale, Multiple sclerosis, Sjögren’s Syndrome

^d^ NMDAR (2), GABA_B_R (1)

^e^ CN1A/GAD65: SOX1 (3); GAD65: SOX1 (1), ZIC4 (1), MA2/TA (2), HU (1), YO (1)

^f^ cortisone, intravenous immunoglobulin therapy, azathioprin, plasmaexchange, rituximab, Mycophenolat, immune adsorption, cyclophosphamide, basiliximab,

^g^ Defined according to The International League Against Epilepsy (ILAE): failure of a patient’s seizures to respond to at least two antiseizure medications that have been appropriately selected and used for an adequate period of time

^h^ ASM = Anti-seizure medication

^i^ CN1A: prostate cancer (1); GAD65 malignant peripheral carcinomas (prostate cancer (3), breast cancer (1), cervical cancer (1)), and multiple myeloma (1)

^j^ including LE (*n* = 16), focal seizures (*n* = 66), unknown/single seizures (*n* = 5), idiopathic generalized epilepsy (*n* = 1)

### High prevalence of CN1A autoantibodies in anti-GAD65-positive neurological patients

The entire anti-GAD65-positive cohort (*n* = 118) was then analysed for the presence of CN1A autoantibodies using specific blots to detect full-length CN1A protein (Fig. 1D) as well as cell-based assays (Fig. 1G). In total, 32 anti-GAD65-positive patients were also positive for CN1A autoantibodies in sera and in available corresponding CSF samples (Fig. 1E), indicating the presence of anti-CN1A autoantibodies in the CNS. Twenty-six patients were positive in both established screening assays, and three were positive for only one of the tests. The prevalence of CN1A autoantibodies was higher in anti-GAD65-positive patients compared to healthy individuals (Fig. 1F). Interestingly, anti-CN1A was also observed in two patients with onconeuronal autoantibodies, in 6/97 samples from age-/sex-matched seronegative patients with clinical suspicion of LE [18] but without reactivity on immunoblot screening [20], and in two healthy donors (2/60).

### Anti-GAD65-positive patients with coexisting CN1A autoantibodies have a higher prevalence of definite limbic encephalitis

We next evaluated the clinical characteristics of the 118 anti-GAD65-positive patients with (*n* = 32) and without CN1A autoantibodies (*n* = 86) which can be found in Table 1. Interestingly, the prevalence of patients for LE at disease onset [18] was significantly higher in the CN1A/GAD65 antibody-positive group (8/32) compared to only anti-GAD65-positive patients (8/86). Overall, 84.4% of the CN1A/GAD65 antibody-positive patients had a typical neurological syndrome known to be associated with GAD65 autoantibodies (27/32). Although autoantibodies against CN1A are known to be a biomarker for sporadic inclusion body myositis (IBM) and are widely detected in other autoimmune diseases (e.g. Sjögren’s syndrome, or systemic lupus erythematosus), none of the CN1A/GAD65 antibody-positive patients presented such syndrome.

### Neuropsychological assessment shows a negative correlation between anti-GAD65 titre and cognitive performance in CN1A/GAD65 antibody-positive patients

At the time point of antibody testing, neuropsychological performing was assessed in 49 patients (16 anti-CN1A/anti-GAD65 patients, 33 anti-GAD65 patients). There were no performance differences between the two groups by means in IQ, executive function, verbal and figural memory (Pearson´s correlation, all F < 1.0, not significant). There was however a difference in mood (Pearson’s correlation; BDI F = 5.479, *p* = 0.023) with the anti-GAD65 patients showing more often a mild depression. Interestingly, in the CN1A/GAD65 antibody-positive patients, performances on memory performance were negatively correlated with the GAD65 autoantibody titres (verbal learning *r*=-0.524, verbal memory *r* = 0.640, figural recognition performance *r*=-0.766, all *p* < 0.01) whereas in only anti-GAD65-positive patients no correlation with autoantibody titres was observed.

### CN1A shows a dendritic distribution in excitatory and inhibitory neurons

**Fig. 2:**
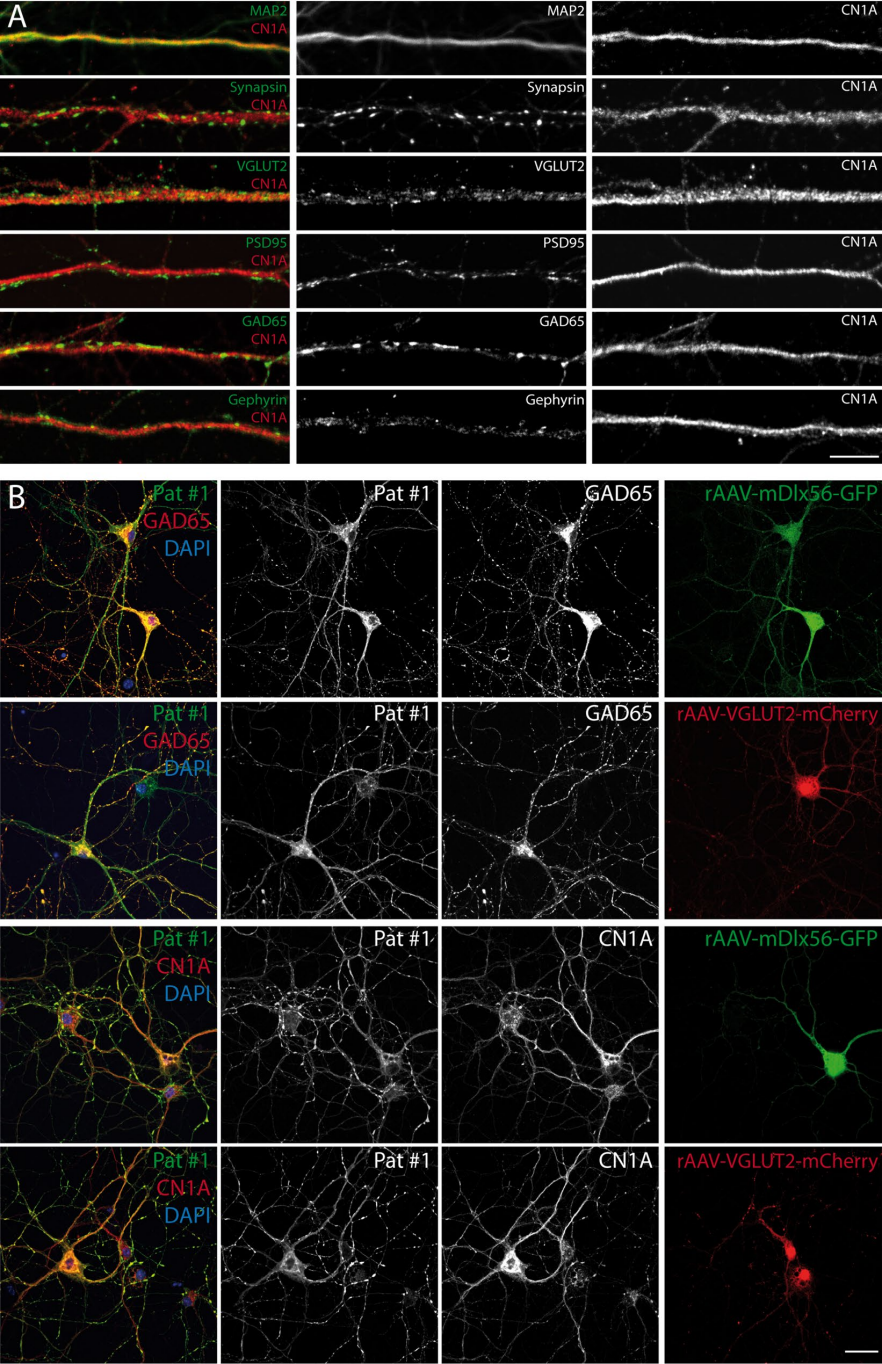
Dendritic non-synaptic localisation of the target antigen CN1A and cellular binding pattern of patient serum on inhibitory and excitatory neurons. (**A**) Primary hippocampal neurons co-immunolabelled with CN1A and markers for dendritic (neuronal dendritic marker: microtubule-associated protein 2, MAP2), pre- (VGLUT2, GAD65) and postsynaptic (postsynaptic density protein 95, PSD95, Gephyrin) proteins of inhibitory and excitatory neurons to determine the localisation of the target antigen CN1A (scale bar 5 μm). (**B**) Binding pattern of patient serum (Pat #1) on fixed cultured neurons at DIV14 shows a reactivity on excitatory (transduced with rAAV-VGLUT2-mCherry; right panel) and inhibitory (transduced with rAAV-mDlx-GFP; right panel) neurons co-localising with both GAD65 and CN1A protein similar to the one observed with the commercial CN1A antibody (scale bar: 50 μm)

At the cellular level, CN1A protein is ubiquitously distributed throughout neuronal dendrites (Fig. 2A, MAP2), of both excitatory (Fig. 2A, excitatory marker: VGLUT2; PSD95) and inhibitory (Fig. 2A, inhibitory markers: GAD65, gephyrin) neurons, but absent from synaptic structures. Consistent with this, incubation of PHN with serum of CN1A/GAD65 antibody-positive patients revealed an immunolabeling pattern in both inhibitory and excitatory neuron types (Fig. 2B).

### Brain region, cell type, and subcellular specific binding pattern of serum from patients with coexisting CN1A/GAD65 autoantibodies

**Fig. 3:**
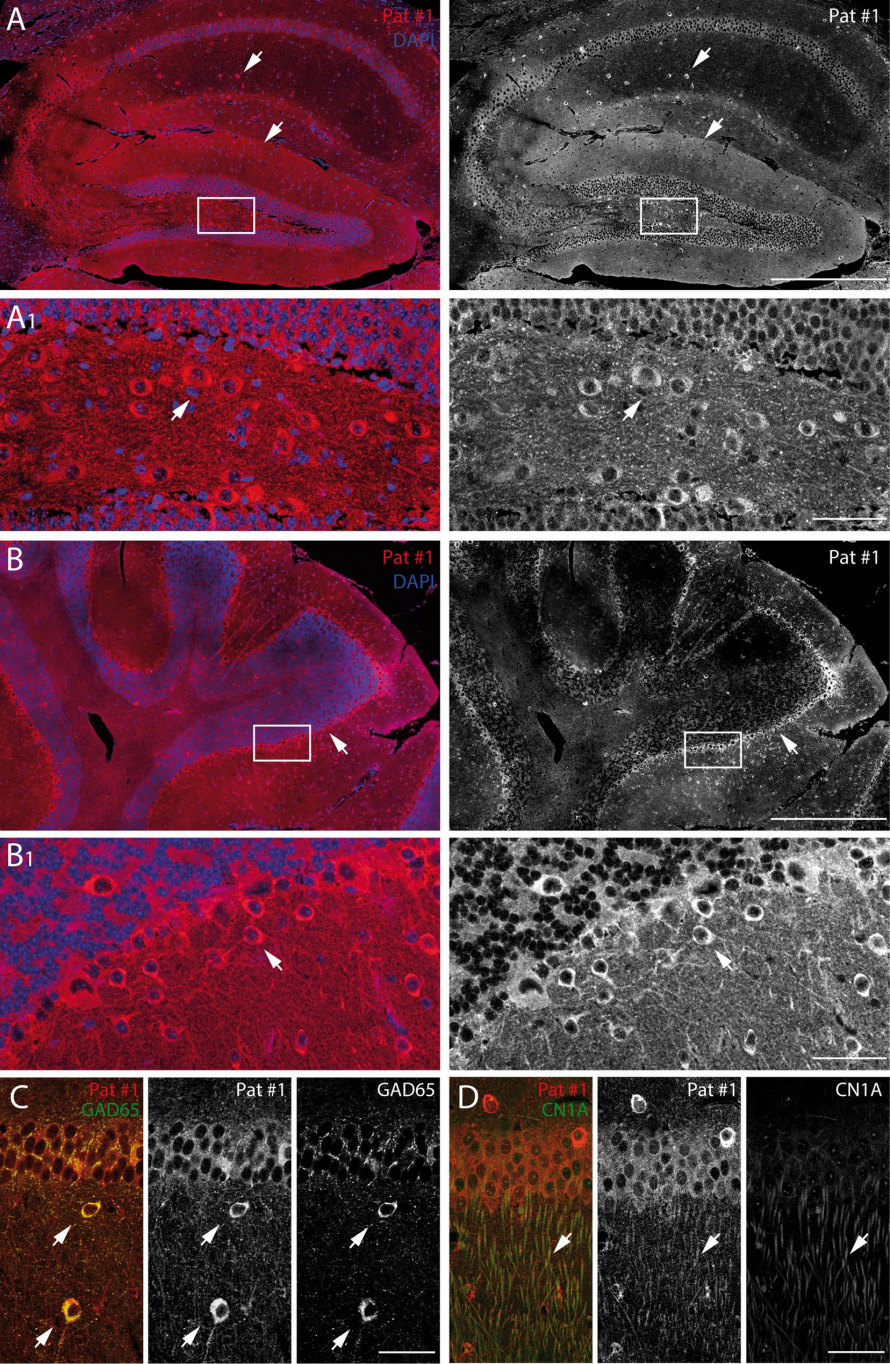
Brain region and cell type specific binding patterns of patient serum with coexisting CN1A/GAD65 autoantibodies and colocalization with GAD65 and CN1A. (**A**) Immunolabelling of patient serum (Pat #1) on mouse brain slices shows an intense pattern of neuropil reactivity in the stratum lacunosum/moleculare of the hippocampal formation, a region with predominantly unmyelinated axons, dendrites and glial cell processes forming a synaptically dense region with only few cell bodies (lower arrow). In addition to labelling of the pyramidal and granular cell layers, a clear labelling of specific single cells in the stratum radiatum (upper arrow) and in the hilar region is present (A1, arrow; scale bars: 500 μm, insert: 25 μm). (**B**) In the cerebellum, patient serum reveals strong binding to inhibitory Purkinje cells (arrows; scale bars: 500 μm, insert: 25 μm). (**C**, **D**) Immunolabelling of mouse brain slices with monoclonal antibodies against GAD65 and CN1A shows a colocalisation of patient serum in (**C**) interneuronal somata and (**D**) dendritic structures (scale bars: 50 μm)

On neuronal structures on mouse brain sections, CN1A/GAD65 antibody-positive patients serum showed an intense reactivity in the hippocampal neuropil (Fig. 3A, lower arrows). In addition to pyramidal and granular cells, there was also a clear labelling of individual cells, mainly in the hilar region (Fig. 3A, A_1_, arrows), but also in the stratum radiatum (Fig. 3A, upper arrows). In the cerebellum, patient serum showed a strong binding to inhibitory Purkinje cells (Fig. 3B, arrows). This cell- and region-specific reactivity of patients’ serum correlates with GAD65 protein in the somata of interneurons (Fig. 3C, arrow) and with CN1A localised mainly in dendritic structures (Fig. 3D, arrow), similar to the target antigens of the autoantibodies.

### Intracellular dendritic uptake of GAD65 and CN1A antibodies into interneurons leads to increased death of cultured neurons

**Fig. 4:**
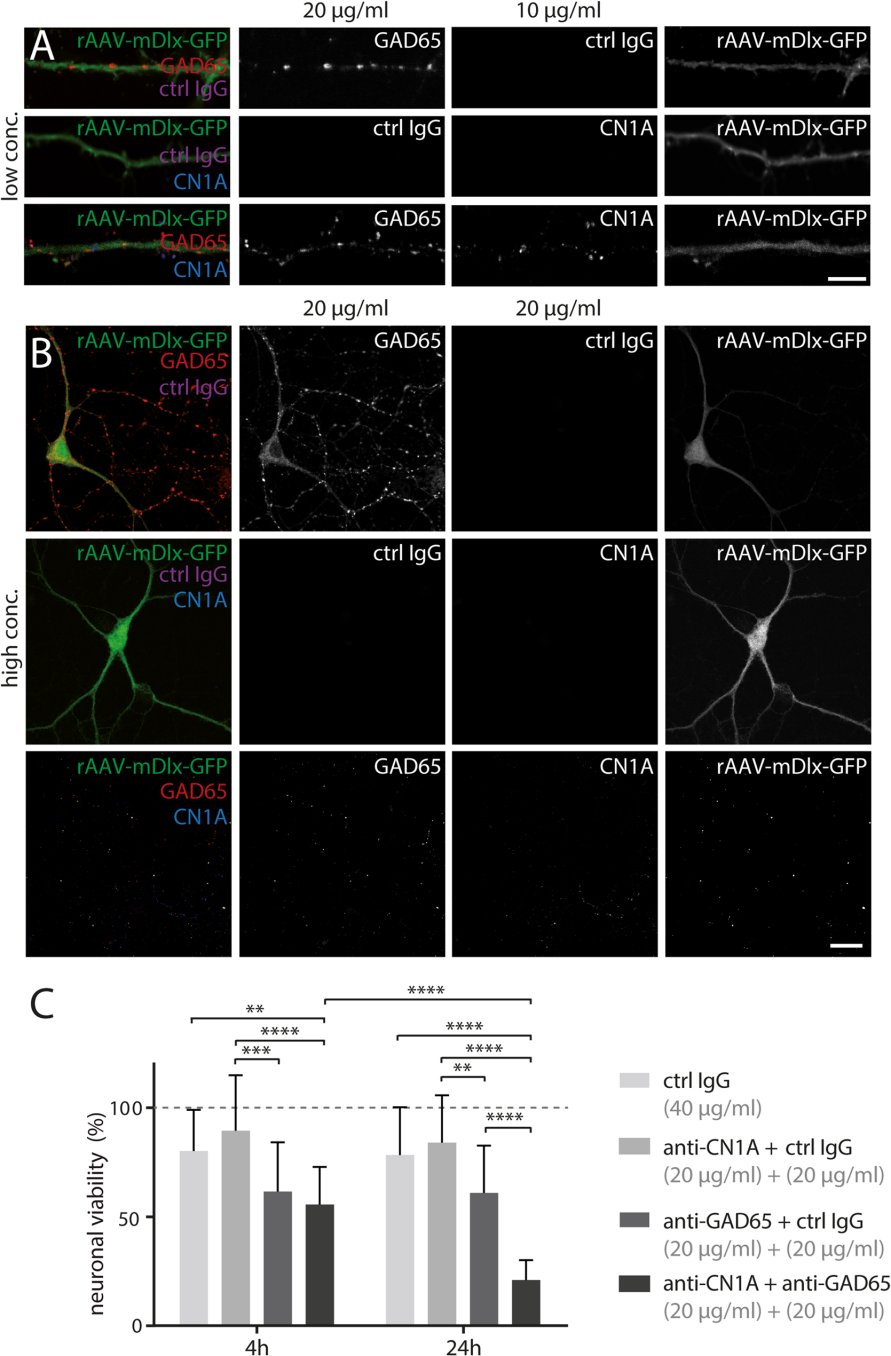
Combined interneuronal uptake of GAD65 and CN1A antibodies results in enhanced neuronal cell death in vitro. (**A**-**B**) Incubation of live hippocampal neurons with GAD65, CN1A antibodies and human control IgG (ctrl IgG) in different combinations for 24 h at 37 °C. Inhibitory neurons were visualized by transduction with rAAV-mDlx-GFP (green) at DIV 4. Intracellular dendritic uptake in hippocampal neurons with different antibody combinations was visualized using the corresponding fluorescently labelled secondary antibody. (**A**) High concentrations of GAD65 antibody (20 µg/ml) and lower concentrations of CN1A antibody (10 µg/ml) were combined or incubated individually. Ctrl IgG was used to keep the total IgG concentration constant (scale bar 5 μm). (**B**) Hippocampal neurons were simultaneously incubated with both CN1A and GAD65 antibodies at high concentrations (20 µg/ml) or each in combination with IgG ctrl (20 µg/ml; scale bar: 50 μm). Ctrl IgG was not taken up into neurons under any condition (**C**) Neuronal viability was quantified after incubation with high antibody/ctrl IgG concentrations (20 µg/ml each) at 4 and 24 h. Values were normalized to native neuronal cultures (*N* = 4 biological replicates, *n* = 16 per group). Data represent results from four separate neuronal cultures with four replicates per condition. Neuronal cell loss was exacerbated by GAD65 and CN1A/GAD65 antibody incubation at both time points (2-way ANOVA, time comparison, *p = 0.014, F_1,75_ = 6.38; condition comparison, ****p < 0.0001, F_4,75_ = 64.18; well comparison: *p* = 0.98, F_75,75_ = 0.62). Significant neuronal loss was observed after 4 h of incubation with anti-GAD65 (38 ± 22%) or CN1A/GAD65 (44 ± 17%) antibodies. More remarkably, after 24 h of incubation, GAD65 antibodies led to a similar neuronal loss (39 ± 21%), whereas a combination of CN1A/GAD65 antibodies dramatically reduced neuronal viability to 21 ± 9% (2-way ANOVA, Tukey’s post hoc test: *p < 0.05, **p < 0.01, ***p < 0.001, ****p < 0.0001). Error bars indicate mean ± SD

To assess neuronal uptake of antibodies and their impact on viability, PHN were incubated with varying concentrations of mouse anti-GAD65. High concentrations of anti-GAD65 with control IgG led to specific intracellular uptake into GABAergic neurons (Fig. 4A, upper panel). In contrast, anti-CN1A with control IgG showed no uptake (Fig. 4A, middle panel). A low concentration of anti-CN1A with high anti-GAD65 induced uptake of both antibodies into GABAergic neuron dendrites (Fig. 4A, lower panel).

Notably, incubation with high concentrations of both GAD65 antibody and control IgG resulted in widespread neuronal cell death (Fig. 4C), following its uptake into GABAergic neurons (Fig. 4B, upper panels). More dramatically, simultaneous incubation with both antibodies massively destroyed neurons (Fig. 4B, lower panel, note absence of stained neurons), with increasing neuronal cell loss (Fig. 4C). In contrast, incubation with high concentrations of anti-CN1A with control IgG had no significant effect on neuronal viability (Fig. 4B, middle panel).

### GAD65 and CN1A/GAD65 antibody-positive patient Sera lead to increased neuronal network activity

**Fig. 5:**
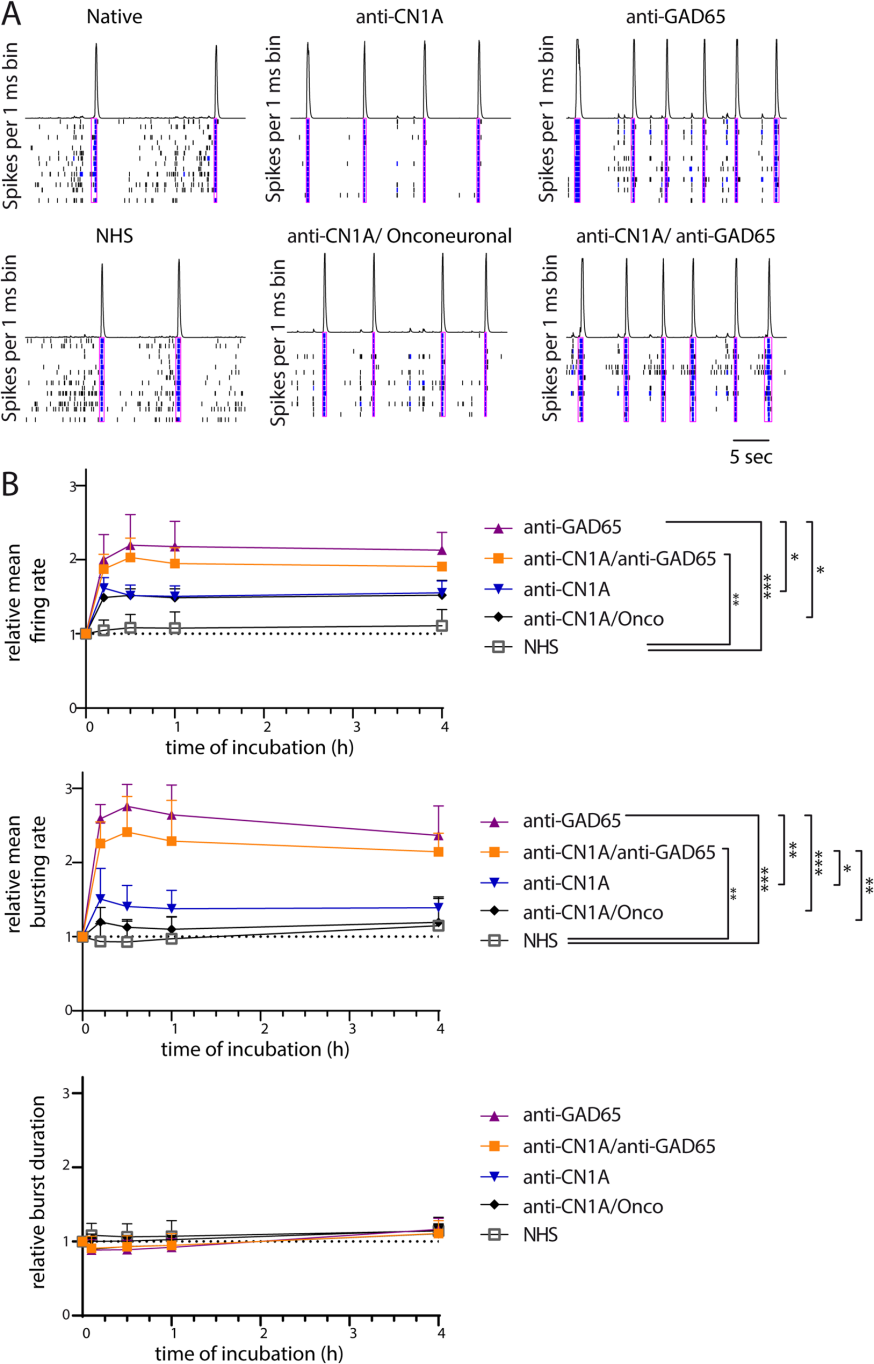
Exposure to CN1A/GAD65 and GAD65-positive sera resulted in increased neuronal network activity in cultured hippocampal neurons. (**A**) Representative spike time histograms of spiking and bursting activity throughout the well after incubation of DIV14 hippocampal neurons incubated with patient sera positive for autoantibodies against CN1A, GAD65, CN1A and GAD65 or CN1A and onconeuronal (oncon) autoantibodies (MA2/TA, CV2, AMPH) as well as untreated native neurons and NHS control recorded with multi-electrode arrays (MEAs). Black lines indicate spikes, blue lines depict burst and magenta rectangles present network bursts. (**B**) Quantification of network activity parameters such as the mean firing rate (total number of spikes divided by the duration of the analysis; upper panel), mean burst firing rate (total number of single-electrode bursts divided by the duration of the analysis; middle panel), and mean network burst firing rate (lower panel) were measured after baseline recording followed by exposure at DIV 14. Data represent results from three separate neuronal cultures. Incubation with GAD65 or CN1A/GAD65 positive sera continuously increased neuronal activity in terms of firing rate as early as 10 min after incubation (2-way ANOVA, time comparison, *p* = 0.524, F_3,30_ = 0.76; group comparison, ****p = 0.0004, F_4,10_ = 14.00; Tukey’s post-hoc: GAD65 vs. NHS: *p* = 0.0004; CN1A/GAD65 vs. NHS: *p* = 0.0017). This effect was absent when incubating with anti-CN1A or anti-CN1A/anti-onconeuronal-positive sera (Tukey’s post-hoc: CN1A vs. NHS: *p* = 0.074; CN1A/oncon vs. NHS: *p* = 0.114). Similarly, the mean burst firing rate was also affected (2-way ANOVA, time comparison, *p* = 0.594, F_3,30_ = 0.64; group comparison, ***p = 0.0001, F_4,10_ = 18.50). In contrast, mean burst duration (total duration of bursts divided by the number of bursts) does not differ between groups, but the time of measurement does have an effect (2-way ANOVA, time comparison, ****p < 0.0001, F_3,30_ = 87.61; group comparison, *p* = 0.743, F_4,10_ = 0.490). Data were normalized to the averaged native baseline recording of each condition. 2-way ANOVA, Tukey’s post hoc test: *p < 0.05, **p < 0.01, ***p < 0.001, ****p < 0.0001. Error bars indicate mean ± SD

To analyse the effects of autoantibody-positive patient sera (anti-GAD65, anti-CN1A, anti-CN1A/anti-onconeuronal, and anti-CN1A/anti-GAD65) on neuronal network activity, in vitro neuronal network activity (ivNNA) was measured on PHN by using MEAs. ivNNA reflects intrinsic network properties in the absence of extrahippocampal sensory inputs. We observed a more synchronized activity pattern after incubation with patient-derived autoantibody-positive sera, with more frequent bursts of longer duration (Fig. 5A). Representative raster plots display this increased frequency of network bursts for CN1A or CN1A/onconeuronal antibody incubation compared to controls.

Only patient-derived sera positive for anti-GAD65 or anti-CN1A/anti-GAD65 strongly increased the activity of hippocampal neurons inducing an elevated mean firing rate that was able to induce a continuously altered coordinated activity pattern (Fig. 5B, top), indicated by an elevated relative mean burst rate over the entire recording period (Fig. 5B, middle). These data suggest that GAD65 autoantibodies acutely elevate coordinated network activity and neuronal hyperexcitability.

### CN1A/GAD65 antibodies induce enhanced autophagy levels in vitro

**Fig. 6:**
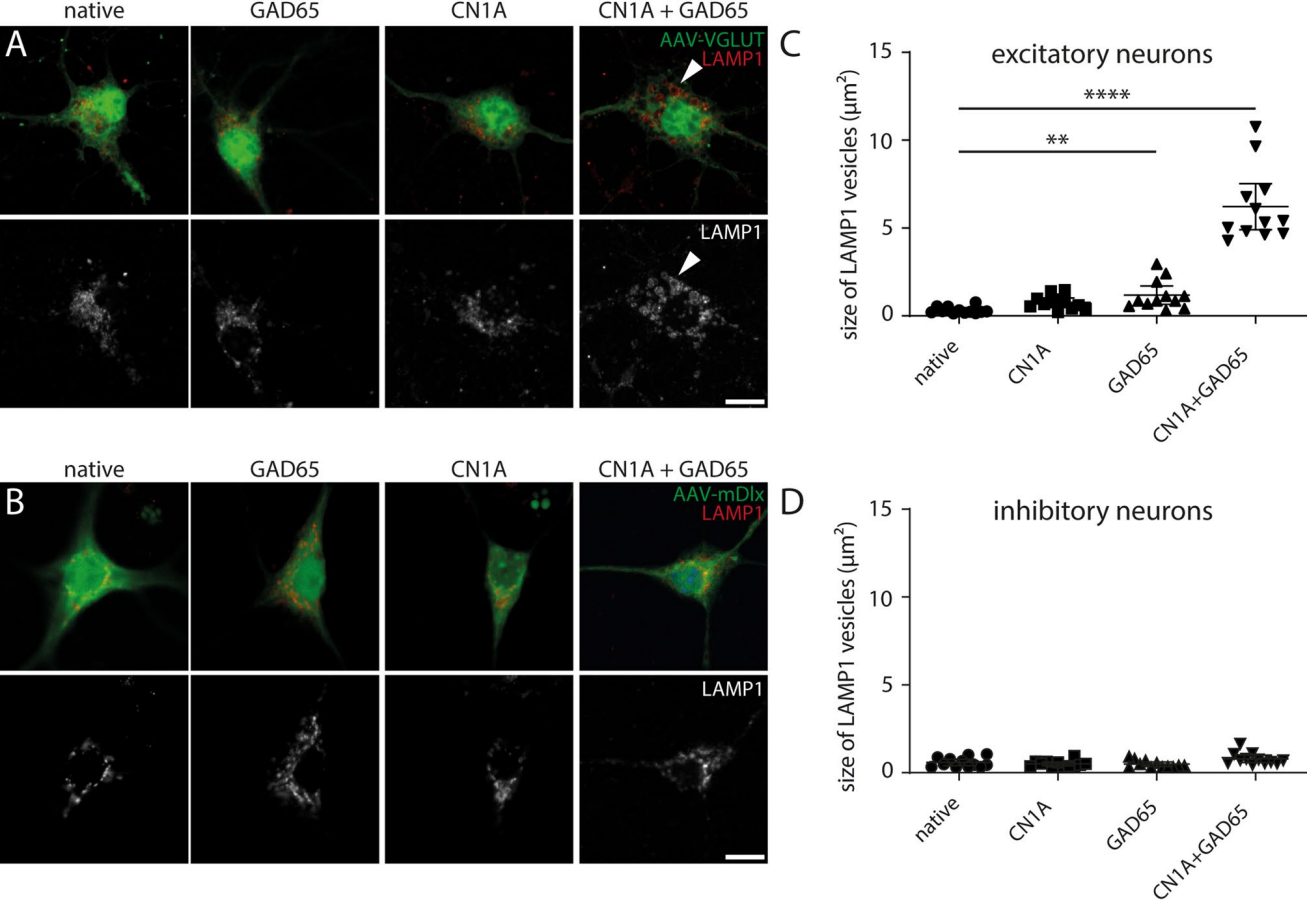
Incubation with CN1A/GAD65 antibodies induces lysosomal enlargement in neuronal cell bodies. (**A**-**B**) Representative confocal images of cultured hippocampal neurons (excitatory: transduction with rAAV-VGLUT2-mCherry; inhibitory: transduction with rAAV-mDlx-GFP, both green) stained with the lysosomal marker LAMP1 (red). (**C**) In excitatory neurons, lysosome size (vesicle area; three independent experiments) is dramatically increased after 24 h incubation with combined CN1A and GAD65 antibodies compared to native neurons (*N* = 3 biological replicates, *n* = 12 wells per group; *n* = 10 lysosomal vesicles per well), whereas single incubation has no significant effect (Kruskal-Wallis test: ****p < 0.0001; Dunn’s post hoc test: native vs. CN1A *p* = 0,0862; native vs. GAD65 **p = 0.009; native vs. CN1A + GAD65 ****p < 0.0001). (**D**) While all groups were slightly different with respect to the size (area) of neuronal lysosomes in interneurons (Kruskal-Wallis test: *p = 0.021; *N* = 3 biological replicates, *n* = 12 wells per group; *n* = 10 vesicles per well), there was no significant increase when compared to native neurons (Dunn’s post hoc test: native vs. CN1A *p* > 0,999; native vs. GAD65 *p* = 0.862; native vs. CN1A + GAD65 *p* = 0.192). No significant changes were found in interneurons when comparing the group effects

To investigate potential effects of CN1A and GAD65 antibody exposure on autophagy processes, we examined lysosomal enlargement in mouse hippocampal neurons by using LAMP1 as a lysosomal marker. After 24 h of antibody incubation, pronounced lysosomal protein enrichment was present exclusively in excitatory neurons exposed to a combination of CN1A/GAD65 antibodies (Fig. 6A, rightmost). In excitatory neurons, the average size (area) of these lysosomes was significantly enlarged by incubation with GAD65 antibody alone and GAD65/CN1A antibodies (Fig. 6C), whereas the size in interneurons was less affected (Fig. 6B-D).

## Discussion

### Coexisting autoantibodies

GAD65 autoantibodies can be observed at high titres in patients with a clinical spectrum of neurological syndromes encompassing stiff-person syndrome, cerebellar ataxia, LE, and drug-resistant TLE. Overlapping symptoms are frequently observed. Our anti-GAD65 positive patients consistently exhibit high-titre antibody levels and were either CBA- and/or line blot-positive. These detection methods are highly specific, semi-quantitative assays that do not detect the low antibody concentrations typically observed in individuals with T1DM or healthy controls [14]. Given that assay positivity corresponds to antibody levels of ≥ 1,400–1,800 U/ml when benchmarked against radioimmunoassay standards, all included patients meet the threshold for high-titre anti-GAD65 seropositivity associated with neurological disease [12, 13]. All patients included in this cohort presented with a well-characterized neurological phenotype.

Coexisting autoantibodies have been discussed as a reason for the diverse disease manifestation [10], but the coexistence of neuronal autoantibodies have only been described in individual case studies (for review [11]). Accordingly, in the present cohort of 118 anti-GAD65-positive neurological patients, autoantibodies against antigens typically found in the context of autoimmune encephalitis were identified in only 11 patients. Thus, based on our systematic analysis, the concept that the presence of autoantibodies identified in other forms of autoimmune encephalitis plays a major role in anti-GAD65-positive patients cannot be verified.

### Coexisting CN1A autoantibodies

Interestingly, a study of anti-GAD65-positive patient sera on hippocampal cultures revealed co-existing autoantibodies against three unknown targets [10]. However, the identity of these co-existing autoantibodies could not be determined. Systematic screening of our cohort of 118 anti-GAD65-positive neurological patients for unknown coexisting autoantibodies revealed a high incidence of 27% to be positive for autoantibodies against the protein cytosolic 5‘-nucleotidase (CN1A), an enzyme that dephosphorylates nucleoside monophosphates and inactivates nucleoside analogues [22], and plays a pleiotropic role in several cellular processes [23]. Gene silencing studies demonstrated that a decrease in CN1A expression activates the apoptotic pathway leading to massive cell death [23].

Most of the CN1A-positive GAD65 patients were positive in both established screening assays (26/32), only three individuals showed positivity in only one of the tests. This finding highlights the complementary nature of both assays and the importance of considering epitope binding characteristics, antibody concentration and methodological differences when detecting autoantibodies against intracellular antigens. CBAs preserve the native conformation of antigens, allowing detection of antibodies that bind conformational epitopes that may not be accessible in the denatured state used in WB. In addition, CBA may be more sensitive to lower concentrations of antibodies that are difficult to detect by WB. In contrast, WB is particularly effective at detecting higher levels of antibodies directed against linear epitopes [18, 24, 25]. The fact that some patients were positive in only one of the assays suggests that each method captures different aspects of antibody recognition and highlights the benefit of using both CBA and WB for a comprehensive assessment of autoantibodies in patients with neurological disorders.

CN1A autoantibodies have previously been reported to be highly specific for sporadic IBM, which is a progressive degenerative muscle disease of autoimmune origin, suggesting that they play a pathogenic role by interfering with protein degradation [26]. CN1A autoantibodies have also been found in other autoimmune diseases, including Sjögren’s syndrome and systemic lupus erythematosus [27].

Clinically, the prevalence of patients in our cohort fulfilling the criteria for definite LE at disease onset [18] was significantly higher in the anti-CN1A/anti-GAD65-positive group compared to patients with anti-GAD65 alone. Interestingly, no anti-CN1A-positive patient had any of the typical anti-CN1A-associated autoimmune diseases described above. Testing for anti-CN1A in patients with intracellular onconeuronal autoantibodies, was positive in 2/32 (6%) of patients.

Functionally, CN1A autoantibodies may induce protein degradation by inhibiting the CN1A enzyme. This mechanism is not limited to neurological disorders, and thus supports the hypothesis that CN1A autoantibodies are not disease specific and are involved in the pathogenesis of various immunological processes. From a clinical perspective, patients who test positive for anti-GAD65 should also be screened for diseases associated with anti-CN1A. An evaluation of anti-CN1A analysis to the broader range of known anti-GAD65 autoantibody-associated autoimmune diseases (e.g. vitiligo, T1DM, Hashimoto’s thyroiditis) may also be considered [28, 29]. This study may also shed some light on the known association between systemic autoimmune diseases and the occurrence of epilepsy [30].

### Localisation of CN1A

CN1A is predominantly expressed in peripheral tissue, but also in other organs, with lower levels in the brain [22], though its neuroanatomical and subcellular distribution remains unclear [31]. Previous studies have reported strong 5’-nucleosidase expression in the some specific hippocampal synaptic regions [32]. In our study, CN1A was ubiquitously expressed in mouse brains, localised in both excitatory and inhibitory neurons, particularly in dendritic structures, suggesting a potential intracellular regulatory role.

### Intracellular antibody uptake

Intriguingly, we have observed that anti-GAD65 antibodies can enter interneurons and reach their intracellular target antigen in hippocampal cell cultures. Here, we further demonstrate a concentration-dependent intracellular neuronal uptake of both CN1A and GAD65 antibodies in parallel. The concentration-dependent uptake of anti-GAD65 into GABAergic neurons appears to trigger the cotransport of other intracellularly binding antibodies, in this case CN1A antibodies, which do not enter the cell when exposed individually. Possible mechanisms are either endocytosis [33] or extracellular antigen presentation [34] followed by subsequent antibody binding and uptake, thereby triggering neurodegeneration and hyperexcitability. Recently, specific cellular uptake via the polymeric immunoglobulin receptor and transcytosis of dimeric immunoglobulin (Ig)A by epithelial cancer cells has been described as a potential new mechanism of cellular Ig uptake [35]. To date, there are conflicting data regarding the pathogenetic significance of human anti-GAD65. While synaptic uptake via Fc receptor-mediated endocytosis and disruption of GABAergic signalling have been observed for other autoantibodies against intracellular antigens [36], this has not yet been demonstrated for GAD65 autoantibodies [13]. As GAD65 forms complexes with the heat shock protein 70 family, it becomes membrane-associated with synaptic vesicles [34] and may thus form a vesicular complex that initiates extracellular presentation of GAD65 at synaptic terminals [37]. This could allow interaction between the autoantibody and antigen at the cell surface and subsequent intracellular uptake leading to functional impairment. Alternatively, these antibodies may enter the dendrite during synaptic vesicle fusion, which is associated with presynaptic neurotransmitter release, and bind to the intracellular protein, similar to amphiphysin autoantibodies [38]. Accordingly, in vivo studies have detected GAD65 monoclonal antibodies in CA1 interneurons shortly after their hippocampal injection [39], suggesting that neuronal uptake is a reasonable scenario.

Experimental active immunization with GAD65 protein has failed to produce a clinical phenotype [40]. However, GAD65 autoantibodies have been shown to inhibit the enzymatic activity of GAD65 in vitro [17], which could have an effect of GAD65 antibodies on GAD function through to misdirected production of GABA. The clinical picture of GAD65 antibody-associated neurological disorders is consistent with a disruption of GABAergic signalling.

Possible explanations for these conflicting in vitro results could be variations in antibody concentration, duration of application, and cell type composition of the cultured neurons, or they could indicate significant differences in tissue distribution or epitope specificity of anti-GAD65 reactivity.

### Autophagy, cell death and hyperexcitability

A recent study in sporadic IBM showed that CN1A autoantibodies affect protein degradation, potentially influencing autophagy and apoptosis [26]. Autophagy regulates cell growth, survival, death, and macromolecule degradation, and is linked to aging, inflammation, and immunity. In neurons, it aids in clearing damaged intracellular components while modulating excitability and synaptic plasticity [41]. Our data show that co-incubation of hippocampal neuronal cultures with both antibodies (anti-CN1A/anti-GAD65) induces hyperexcitability, lysosomal enlargement in excitatory neurons, and severe neuronal death– effects surpassing those of anti-GAD65 alone– whereas anti-CN1A alone shows no significant impact. Neuronal damage from high intracellular levels of anti-CN1A resembles catabolic/autophagic effects observed in IBM [26] and may induce a vicious cycle of autophagic destruction leading to neuronal death. Unlike anti-Drebrin autoantibodies found in TLE patients [21], anti-GAD65 does not alter postsynaptic dendrite signal length, likely due to GAD65’s localisation at axon terminals.

Given that GAD65’s enzymatic activity is tightly regulated by binding to its co-factor pyridoxal 5′-phosphate [42], anti-CN1A may interfere by dephosphorylating this co-factor through its nucleotidase function, disrupting GABA homeostasis. Additionally, anti-CN1A antibodies may impair autophagic lysosomal degradation, facilitating apoptosis. The accumulation of GAD65 autoantibodies may further reduce GABA synthesis [17], causing imbalance and excitotoxicity. In the hippocampus, reduced inhibitory interneuron activity may lead to overall network hyperexcitability and neuronal death [43].

## Conclusion

**Fig. 7:**
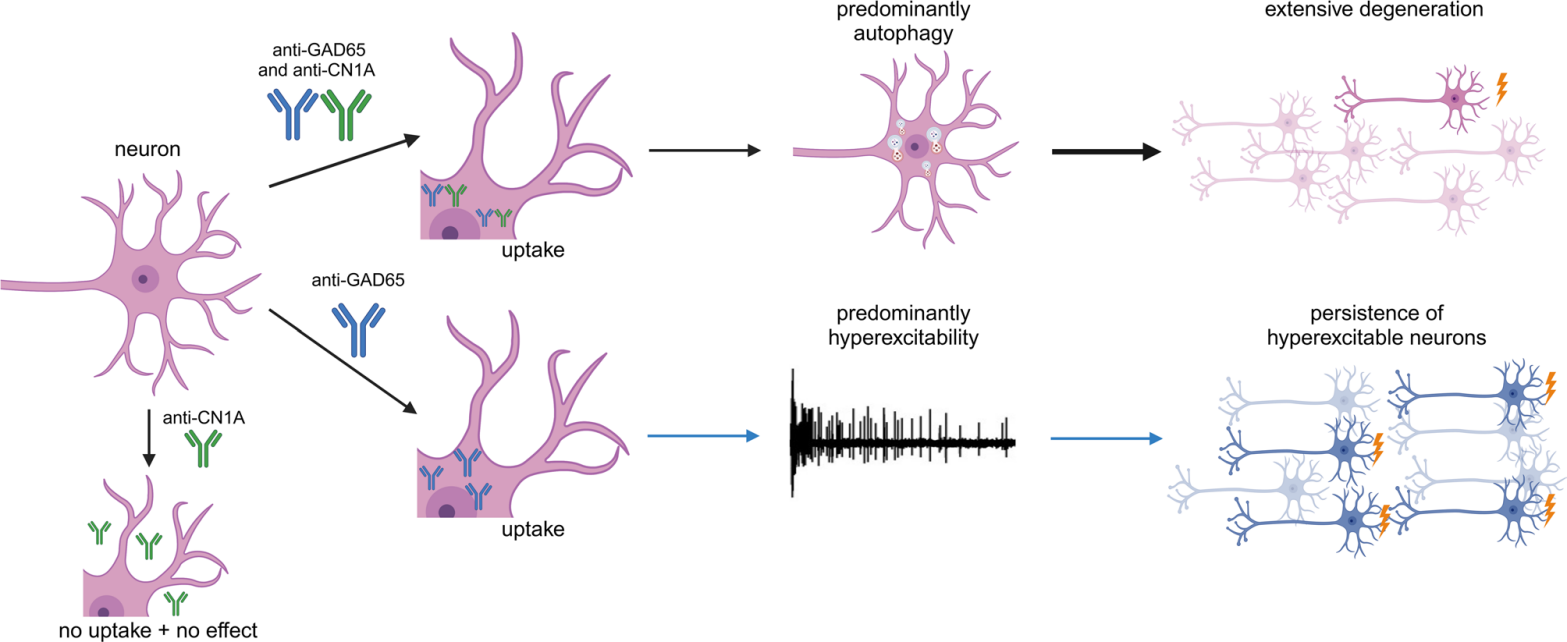
Graphical abstract: Functional pathological effects of additional autoantibodies against CN1A that enhance the response of GAD65 autoantibodies. GAD65 autoantibodies increase neuronal network activity but neuronal uptake of anti-CN1A combined with anti-GAD65 leads to aggravated neuronal cell death associated with enhanced autophagy. Created with Biorender

We report on neurological patients with GAD65 autoantibodies who also present with CN1A autoantibodies. While anti-GAD65 has an independent pathogenic potential, CN1A autoantibodies appear to play a pivotal role in exacerbating anti-GAD65-mediated pathology by enhancing intracytoplasmic effects (Fig. 7). The co-occurrence of these autoantibodies in GAD65-positive patients reveals a previously unrecognized mechanism of disease progression and may contribute to the clinical diversity with important implications for future therapeutic implications.

Our data suggest that patients positive for anti-GAD65 should be screened for anti-CN1A-associated diseases, and that a more comprehensive evaluation of anti-CN1A in anti-GAD65-associated autoimmune diseases (e.g. T1DM, vitiligo, Hashimoto’s thyroiditis) may clarify the links between systemic autoimmunity and epilepsy.

## Electronic supplementary material

Below is the link to the electronic supplementary material.

Supplementary Material 1

Supplementary Material 2

## Data Availability

No datasets were generated or analysed during the current study.
