# Supplementary material for: Coexistence of CN1A autoantibodies in GAD65 encephalitis exacerbates neurodegeneration: Novel autoantibodies in GAD65 encephalitis
Source: J Neuroinflammation. 2025 Jul 26;22:194. doi: 10.1186/s12974-025-03521-4 (PMC12297844; doi:10.1186/s12974-025-03521-4)
Supplement: Supplementary file 1 — Supplementary Material 1 [file 12974_2025_3521_MOESM1_ESM.docx]

**Supplementary Material – Blots and gels**

**Coexistence of CN1A autoantibodies in GAD65 encephalitis exacerbates neurodegeneration**

Delara Kamalizade^1^, Annika Breuer^1^, Tobias Baumgartner^1^, Juliane L. Berns^1^, Thoralf Opitz^2^, Franziska S. Thaler^3,4^, Susanne Schoch^1,5^, Lars Komorowski^6^, Christoph Helmstaedter^1^, Rainer Surges^1^, Albert J. Becker^5#^, Julika Pitsch^1#^

Related to Figure 1A:

Immunoblot (IB)

Patient #1


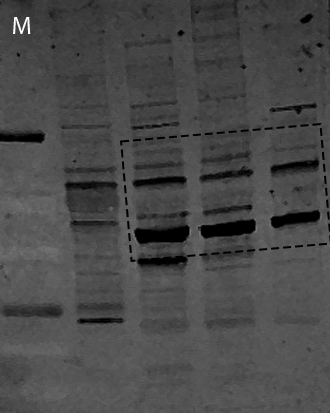


Immunoblot (IB)

Patient #2


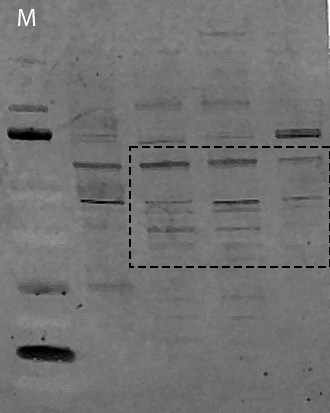


Original images acquired with the Odyssey CLx (Li-Cor) of representative immunoblots (cropped parts shown in Figure 1A) of immunoblots on brain lysates of rat and mice brains incubated with patient-derived serum samples.

1^st^ line (M) = Bio Rad, 161-0374, Precision Plus Protein Dual Color Standard.

Related to Figure 1B:

Immunoblot (IB)

M Pat #1 ctr


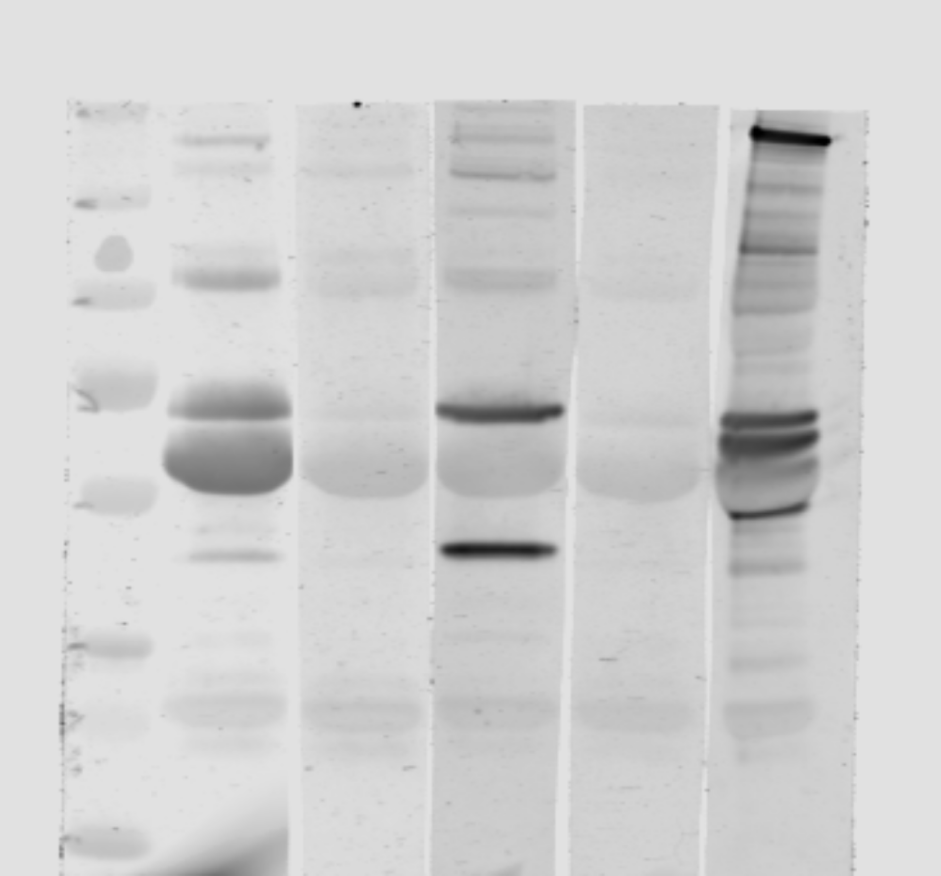


Original images acquired with the Odyssey CLx (Li-Cor) of representative immunoblots (cropped parts shown in Figure 1B) of immunoblots on brain lysates of mice brains incubated with patient-derived serum samples. Please note: As the blots are incubated with individual serum, these blots must be cut before incubation with the serum. M = Thermo Scientific™ PageRuler™ Plus Prestained Protein Ladder, 10 bis 250 kDa.

Coomassie

M Pat #1


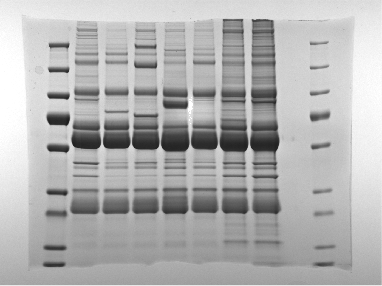


Original images acquired with the Odyssey CLx (Li-Cor) of representative immunoblots (cropped parts shown in Figure 1B) of Coomassie gel on brain lysates of mice brains incubated with patient-derived serum samples. M = Thermo Scientific™ PageRuler™ Plus Prestained Protein Ladder, 10 bis 250 kDa.

Related to Figure 1D:


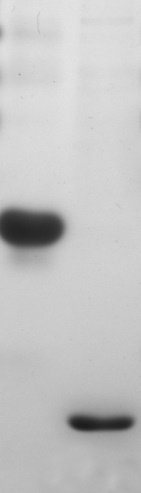

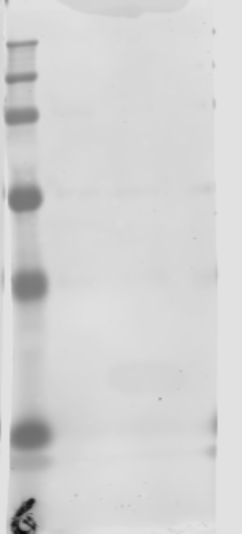

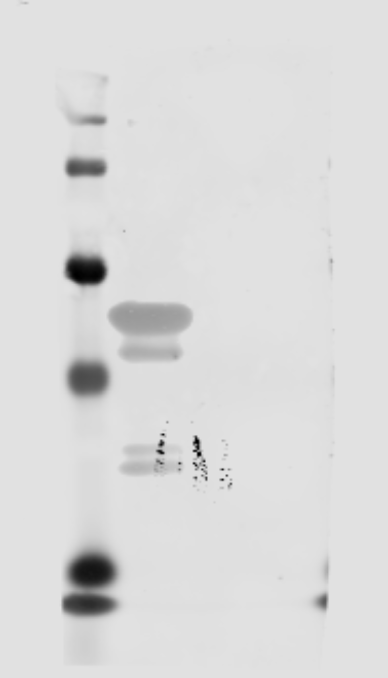

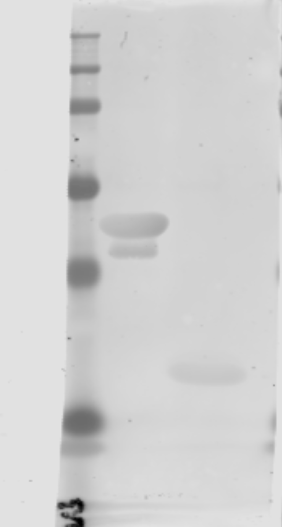

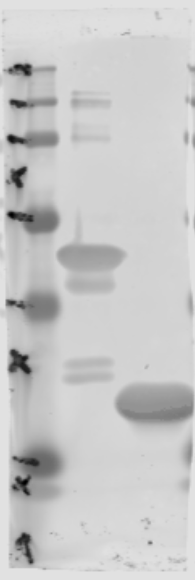


Coomassie neg. ctr commercial anti-CN1A Pat #1 Pat #3

Original images acquired with the Odyssey CLx (Li-Cor) of representative immunoblots (cropped parts shown in Figure 1A) of immunoblots on human Drebrin protein incubated with patient-derived serum samples.

M = Thermo Scientific™ PageRuler™ Plus Prestained Protein Ladder, 10 bis 250 kDa.

Related to Figure 1E:


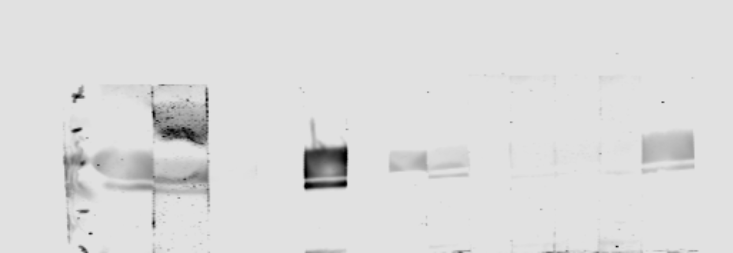


M Pat #1 neg Pat

Pat #3

Original images acquired with the Odyssey CLx (Li-Cor) of representative immunoblots (cropped parts shown in Figure 1A) of immunoblots on human Drebrin protein incubated with patient-derived CSF samples. Please note: As the blots are incubated with individual serum, these blots must be cut before incubation with the serum. M = Thermo Scientific™ PageRuler™ Plus Prestained Protein Ladder, 10 bis 250 kDa.
