# Supplementary material for: Coexistence of CN1A autoantibodies in GAD65 encephalitis exacerbates neurodegeneration: Novel autoantibodies in GAD65 encephalitis
Source: J Neuroinflammation. 2025 Jul 26;22:194. doi: 10.1186/s12974-025-03521-4 (PMC12297844; doi:10.1186/s12974-025-03521-4)
Supplement: Supplementary file 2 — Supplementary Material 2 [file 12974_2025_3521_MOESM2_ESM.docx]

**Supplementary Material and Methods**

**Coexistence of CN1A autoantibodies in GAD65 encephalitis exacerbates neurodegeneration**

Annika Breuer^1§^, Delara Kamalizade^1§^, Tobias Baumgartner^1^, Juliane L. Berns^1^, Thoralf Opitz^2^, Franziska S. Thaler^3,4^, Susanne Schoch^1,5^, Lars Komorowski^6^, Christoph Helmstaedter^1^, Rainer Surges^1^, Albert J. Becker^5#^, Julika Pitsch^1#^

**Autoantibody diagnostics**

**Semi-quantitative immunoblotting and cell-based indirect immunofluorescence testing**. With commercial kits we tested for onconeuronal autoantibodies targeting AMPH, CV2, PNMA1/2 (MA2/TA; paraneoplastic antigen Ma), RI (antineuronal nuclear antibody 2; ANNA2), YO (PCA-1,Purkinje cell cytoplasmic antibody type 1), HU (antineuronal nuclear antibody 1; ANNA1), SOX1 (Sry-like high mobility group box1), ZIC4 (Zinc finger protein Zic4), and GAD65 using semi-quantitative immunoblots according to the manufacturer’s protocols (EUROLINE PNS 12, Euroimmun, DL 1111-1601-7 G) with serum diluted in commercial buffer 1:100, CSF undiluted. Complementary, indirect immunofluorescence (IIFT: ‘Autoimmune-Enzephalitis-Mosaik1’, Euroimmun, FA 1120-1005-1; GAD65-IIFT, Euroimmun, FA 1022-1005-50) was applied relying on HEK293-cells (Human Embryonic Kidney 293) overexpressing the individual cell-surface antigens: NMDAR (N-methyl-D-aspartate receptor), CASPR (contactin-associated protein-like 2), LGI1 (Leucine-rich, glioma inactivated 1), GABA_B_R (gamma-aminobutyric acid receptor B), AMPAR (α-amino-3-hydroxy-5-methyl-4-isoxazolepropionic acid receptor), or GAD65, following the manufacturer’s protocol described in more detail elsewhere (1) (dilution in Phosphate buffered saline with 0.2% Tween 20 (PBST): serum 1:10; CSF undiluted).

All serum samples of seronegative and healthy control individuals were negative for common neurological autoantibodies using above mentioned commercial kits for diagnostic procedures.

**Human samples.** The clinical parameters for the respective groups were: GAD65-positive patients (n=118): male: n=41; female: n=77; median age at onset: 43.5, range: 2-79 y; median age at diagnosis: 48.5, range: 2-79 y; Neurological patients with specific onconeuronal autoantibodies (n=32): male: n=15; female: n=17; median age: 48, range 10-78 y; with either MA2/TA (PNMA1/2; paraneoplastic antigen Ma; n=11), CV2 (CRMP, collapsin response mediator protein 5; n=14), or AMPH (Amphiphysin; n = 7); Age-/sex-matched seronegative neurological patients (n=97): male: n=51; female: n=46; median age: 54, range 9-89 y); healthy donors (n=60): male: n=28; female: n=32; median age: 28, range 21-66 y

**Immunoprecipitation and mass spectrometry.** Freshly dissected mouse brain tissue (1 g) was homogenized in 5ml ComplexioLyte 47a buffer (Logopharm) containing protease inhibitors (Sigma-Aldrich, P8340; Calbiochem, 539134). Lysates were incubated for 3 h at 4°C and centrifuged at 21000xg for 15 min at 4°C. The supernatant was retained and incubated with anti-GAD65-positive patient or control serum overnight at 4°C. Protein G Dynabeads (ThermoFisher, 10004D) were added and incubated for 3 h at 4°C. The beads were washed and eluted at 70°C for 10 min using NuPAGE^TM^ LDS sample buffer (ThermoFisher, NP0007) containing 25 mM Dithiothreitol (Sigma, R0861), alkylated with 60mM IAA (Sigma, I1149) for 30 min in the dark and loaded on 4-12% SDS-PAGE gels (NuPAGE^TM^ system, ThermoFisher, NP0335BOX). Bands that were only observed in patient samples but not in healthy control sera were excised from the gel. Analogue sections of the same size were cut from the control samples.

**Validation of CN1A autoantibody binding**. To establish a screening assay for cytosolic 5’-nucleotidase 1A (CN1A), we used purified recombinant human CN1A protein (kindly provided by Euroimmun, Lübeck, Germany). Immunoblotting with the serum of both index patients showed a band with an expected size of 40-kDa. CN1A protein (110 ng) was loaded onto an SDS-PAGE gel and either stained with Coomassie Brilliant Blue (ROTI Blue, Roth, A152.1) or blotted on an 0.45 µm nitrocellulose membrane. Blotting was performed overnight at 45 mA and 4°C in transfer buffer (25 mM Tris, 192 mM Glycine, 20% methanol and 0.0375% SDS in ddH2O). Next day membranes were incubated for 1 h at RT with blocking solution (2% FCS, 2% w/v bovine serum albumin (BSA) in PBS). The membrane was incubated with biomaterial (serum 1:100; CSF 1:2) or commercial CN1A antibody as a positive control (PA5-101545, ThermoFisher, 1:1000) in blocking buffer overnight at 4°C. Bands were visualized using goat anti-mouse and goat anti-human IRDye 800CW (926-32210, 926-32232, Odyssey, 1:25000) in blocking solution. Membranes were incubated with 2nd ABs for 2 h at room temperature (RT) after three 10 min washing steps with TTBS (29.2% w/v NaCl, 0.25%w/v Tris and 0.5% Tween20 in ddH2O). Signals were measured using an Odyssey CLx (Li-Cor).

A cell-based assay (CBA) was performed using HEK 293T cells seeded on poly-D-lysine-coated coverslips in 24-well plates. Cells were cultured in DMEM (10% FCS, 1% pen/strep), then switched to IMDM with 5% FCS three hours prior to transfection with 500 ng pAAV-CMV-huCN1a-His per well using calcium phosphate (250 mM CaCl_2,_ 2x HEBS). After 24 h, cells were fixed with 4 % PFA, permeabilized (0.1% Triton X-100), and incubated overnight at 4°C undiluted serum. Detection of transfected cells was performed with anti-CN1a antibody (ThermoFisher, PA5-101545) and fluorescent secondary antibodies (goat anti-human 488, Invitrogen, A11013, 1:200; goat anti-rabbit 647, Invitrogen, A21244, 1:200; DAPI, 1:100). Imaging was conducted on a Zeiss Axio Vert.A1 (LD A-Plan 20x/0.35).

**Immunocytochemistry.** PHN were transduced at day *in vitro* (DIV) 4-6 with rAAV-mDlx-GFP (expressed in GABAergic neurons (2)) or rAAV-VGlut2-mCherry (vesicular-glutamate transporter 2; expressed in excitatory neurons; plasmid kindly provided by MP Anderson, Harvard University; rAAVs purchased from Virus Core Facility of the Medical Faculty of Bonn University), fixed at DIV14 with 4% paraformaldehyde (PFA) in PBS for 10 min, three times PBS washed, permeabilised with 0.3% Triton X-100 (Sigma-Aldrich) in PBS for 10 min, blocked for at RT (0.1% Triton X-100, 1% BSA and 10% normal goat serum (NGS) in PBS), and incubated with primary antibodies (anti-CN1A, PA5-101545, ThermoFisher, 1:1000; anti-GAD65, ab26113, abcam, 1:1000) or human patients’ sera (1:100) in blocking buffer at 4°C. After washing, respective Alexa Fluor® secondary antibodies (goat anti-human A11013, Invitrogen; goat anti-mouse A11001, Invitrogen 1:1000; goat anti-guinea pig A11-073, Invitrogen 1:1000) and 4′,6-Diamidin-2-phenylindol (DAPI, 1:10.000, Sigma-Aldrich) in PBS/ Triton X-100 were incubated for in blocking buffer, and mounted (Mowiol, Roth).

**Immunohistochemistry.** Immunohistochemistry was performed on 4 µm thick paraffin sections of mouse brains using standard protocols (3). Briefly, brain slices were incubated with CN1A/GAD65 autoantibody-positive patient serum (1:50) or with commercial antibodies: anti-GAD65 (1:200, abcam, ab26113), anti-CN1A (1:200, ThermoFisher, PA5-101545) in blocking buffer overnight at 4°C. After washing (PBS), respective secondary antibody (Alexa^®^Fluor A11001, A11008, A21090, 1:200) and DAPI (1:10.000) were incubated for 2 hours at RT followed by washing and mounting (Mowiol).

**Recording and analysis of network activity in hippocampal neurons.** With multi-electrode arrays (MEA), signals from 16 electrodes per well were recorded simultaneously (Maestro Edge, Axion Biosystems, Atlanta) from PHN (~1800 cells/mm^2^) grown on CytoView-MEA-24-well-plates with a sampling frequency of 12.5 kHz. *In vitro neuronal network activity* (ivNNA) was measured by incubation with anti-CN1A/anti-GAD65, anti-CN1A/onconeuronal antibody (CV2, MA2, AMPH), anti-CN1A, or anti-GAD65-positive patients (pooled samples of n=4) starting on DIV14 compared with normal human serum (NHS; healthy control) and to native cultures. GAD65 autoantibody titre was ≥ 1:3.200. Data was measured at different time points (10, 30, 60 min and 4 h) and recorded with AxIS software (Axion Integrated Studio Navigator 1.5, Axion Biosystems, Atlanta).

**Autophagy approach.** PHN were transduced at DIV4-6 with rAAV-mDlx-GFP or rAAV-VGlut2-mCherry (Virus Core Facility of the Medical Faculty of Bonn University), on DIV13 incubated with antibodies (rabbit anti-CN1A, PA5-101545, ThermoFisher; mouse anti-GAD65, ab26113, abcam) or control IgG in a total concentration of 20 µg/ml for 24 h at 37°C. After washing, PHNs were fixed at DIV14 with 4% PFA in PBS for 10 min, followed by three PBS washing steps, permeabilised with 0.3% Triton X-100 (Sigma-Aldrich) for 10 min, blocked for at RT (0.1% Triton X-100, 1% BSA and 10% NGS), followed by overnight incubation with primary antibody (anti-LAMP1, ab208943, Abcam, 1:400) at 4°C. After washing, Alexa Fluor® secondary antibodies (goat anti-rabbit 647; A21244, Invitrogen; 1:1000) and DAPI (1:10.000) in PBS/Triton X-100 were incubated for 1 h, and mounted (Mowiol, Roth).

1. Kuehn JC, Meschede C, Helmstaedter C, Surges R, von Wrede R, Hattingen E, et al. Adult-onset temporal lobe epilepsy suspicious for autoimmune pathogenesis: Autoantibody prevalence and clinical correlates. PloS one. 2020;15(10):e0241289.

2. Dimidschstein J, Chen Q, Tremblay R, Rogers SL, Saldi GA, Guo L, et al. A viral strategy for targeting and manipulating interneurons across vertebrate species. Nat Neurosci. 2016;19(12):1743-9.

3. Pitsch J, Kamalizade D, Braun A, Kuehn JC, Gulakova PE, Ruber T, et al. Drebrin Autoantibodies in Patients with Seizures and Suspected Encephalitis. Ann Neurol. 2020;87(6):869-84.
